# Involving community pharmacists in interprofessional collaboration in primary care: a systematic review

**DOI:** 10.1186/s12875-024-02326-3

**Published:** 2024-04-01

**Authors:** Morgane Angibaud, Maud Jourdain, Solene Girard, Louise Rouxel, Adam Mouhib, Antoine Nogueira, Cédric Rat, Jean-François Huon

**Affiliations:** 1 Primary Care Federative Department, Faculty of Medicine, Nantes Université, Nantes, France; 2 National Institute for Health and Medical Research, INSERM U1302 Team 2, INCIT, Team 2, Nantes, France; 3 Department of General Practice, Faculty of Medicine, Nantes Université, Nantes, France; 4 Clinical Pharmacy Unit, Faculty of Pharmacy, Nantes Université, Nantes, France; 5 Nantes Université, CHU Nantes, Pharmacie, F-44000 France; 6 UMR INSERM 1246 SPHERE “methodS in Patient-centered Outcomes and HEalth ResEarch, Nantes Université, Université de Tours, Tours, France

**Keywords:** Collaborative practice, Community pharmacist, Primary care

## Abstract

**Background:**

The World Health Organization supports interprofessional collaboration in primary care. On over the past 20 years, community pharmacists had been taking a growing number of new responsibilities and they are recognized as a core member of collaborative care teams as patient-centered care providers. This systematic review aimed to describe interprofessional collaboration in primary care involving a pharmacist, and its effect on patient related outcomes.

**Methods:**

A systematic review of randomized controlled trials cited in the MEDLINE, EMBASE, PsycInfo and CINAHL in English and French was conducted from inception to November 2022. Studies were included if they described an intervention piloted by a primary care provider and included a pharmacist and if they evaluated the effects of intervention on a disease or on patient related outcomes. The search generated 3494 articles. After duplicates were removed and titles and abstracts screened for inclusion, 344 articles remained.

**Results:**

Overall, 19 studies were included in the review and assessed for quality. We found 14 studies describing an exclusive collaboration between physician and pharmacist with for all studies a three-step model of pharmacist intervention: a medication review, an interview with the patient, and recommendations made to physician. Major topics in the articles eligible for inclusion included cardiovascular diseases with blood pressure, diabetes, dyslipidemia, and risk of cardiovascular diseases. Positive effects concerned principally blood pressure.

**Conclusions:**

Collaboration involving pharmacists is mainly described in relation to cardiovascular diseases, for which patient-centered indicators are most often positive. It underscores the need for further controlled studies on pharmacist-involved interprofessional collaboration across various medical conditions to improve consensus on core outcomes measures.

**Supplementary Information:**

The online version contains supplementary material available at 10.1186/s12875-024-02326-3.

## Introduction

The World Health Organization supports interprofessional collaboration in primary care [1]. Interprofessional collaboration is defined as multiple health professionals from different backgrounds working together with patients, their families, carers and communities to deliver high quality patient-centered care [1]. In primary care, interprofessional collaboration has been shown to improve patient pathways, healthcare efficiency and cost-effectiveness [2–4], and job satisfaction for healthcare providers [5, 6].

Delegating healthcare responsibilities to pharmacists in the context of interprofessional collaboration is one aspect of healthcare that has been adapted to meet the increasing demands and needs to access safe and effective healthcare [5, 7]. This became particularly necessary during the COVID-19 pandemic when interprofessional collaboration rapidly developed to support severely challenged healthcare systems worldwide [8]. To address the heightened strain on healthcare resources, several countries have restructured emergency medical services and reassigned healthcare professional responsibilities [9]. This includes the participation of community pharmacists in COVID-19 screening and vaccination [10, 11]. These additional responsibilities further expanded the growing number of new responsibilities that pharmacists had been taking on over the past 20 years. They include screening for human immunodeficiency virus (HIV) [12], diabetes [13], or cancer [14, 15], prescribing medication (initiation, continuation or modification) [16–18] and reviewing and monitoring prescribing guidelines [19].

To date, some reviews have investigated interprofessional collaborations in healthcare [20, 21]. However, little information is available in the literature about the effects of pharmacist involvement in interprofessional collaboration in primary care, and how it is organized. This systematic review aimed to describe interprofessional collaboration in primary care involving a pharmacist, and its effect on disease or patient related outcome.

## Method

This systematic review was performed according to PRISMA guidelines [22], using MEDLINE (Pubmed), EMBASE, PsycInfo and CINAHL from inception to July 2021. Subsequently, an abbreviated MEDLINE search update from July 2021 to November 2022 was performed. This review constitutes a secondary analysis of a systematic review conducted on interprofessional collaborations in primary care [23].

The following search strategy was used in PubMed: (“Intersectoral Collaboration“[Mesh] OR “Cooperative Behavior“[Mesh] OR “Patient Care Team“[Mesh:NoExp]) AND (“Primary Health Care“[Mesh]) AND (“Outcome and Process Assessment, Health Care“[Mesh]). “This search strategy was adapted to the syntax of Embase, PsychINFO, and CINHAL databases: “intersectoral collaboration”, “cooperative behavior”, “patient care team” AND “primary health care” AND “outcome and process assessment, health care”. The details concerning the search strategy were presented in Supplementary material 1.

To be included, a study had to have reported a randomized controlled trial (RCT) evaluating the effects of a pharmacist intervention on a disease or patient related outcome. The intervention had to be in the context of interprofessional collaboration, which was piloted by a primary care provider and published in English or French. Studies were excluded if they did not report interprofessional collaboration and if they were not in IMRAD format.

Four reviewers (SG, LR, AN and AM) screened the titles and abstracts of the database records and retrieved the full texts and independently examined the studies for eligibility. Disagreements were resolved by consensus discussion with three reviewers (MJ, MA, JFH). The reference lists of included studies were hand searched for additional citations. In a second stage, given the large number of articles, only articles involving a pharmacist were selected.

From those studies included, data concerning the study (authors, publication date, country, sample size, objective and pathology, study duration, inclusion criteria), the intervention (professionals involved, intervention type or practice collaboration), and measures to assess the intervention effect (main outcome measure, secondary outcomes) were extracted.

Two independent reviewers (MA, JFH) appraised the risk of bias and quality of each included study using the Integrated Quality Criteria for Review of Multiple Study Designs (ICROMS) tool [24].

The systematic review was registered in PROSPERO under number CRD42021278461. The registered protocol substantially differs from the review methods. We have focused on pharmacist interventions and presented only results on the effects of interprofessional collaboration on patients to enhance clarity. We have increased reviewers from two to four to address the paper volume.”

## Results

**Fig. 1:**
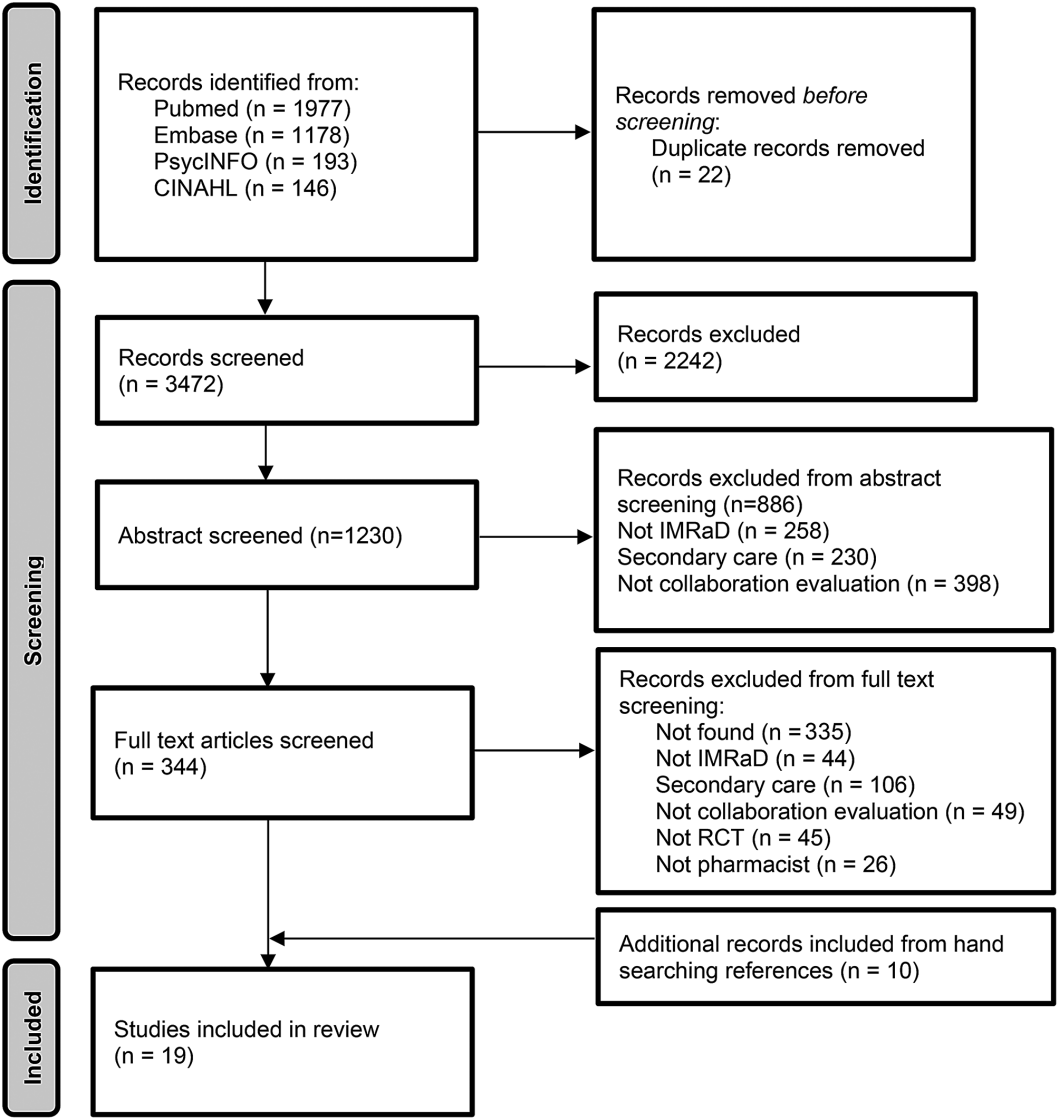
PRISMA Flow Diagram. Abbreviations: IMRaD: Introduction, Methods, Results and Discussion, RCT: randomized controlled trials

Figure 1 presents the PRISMA Flow Diagram. In total, 3472 records were identified of which 2242 were excluded. The excluded studies were interventions delivered by a secondary care professional or did not investigate interprofessional collaboration. Then, 1230 abstracts and 344 full text papers were screened for eligibility and 19 were included in the review.

### Study characteristics

Table 1 lists the study characteristics. Among the 19 studies included, most were performed in North America (*n* = 16), only two in Asia and one in Europe. A majority (*n* = 14) were published in 2009 or later [25–38], the oldest study (Finley et al.) was published in 2002 [39]. Eight studies evaluated a collaborative pharmacist intervention for six months [25, 28, 32, 36, 37, 39–41] and eight studies for more than 12 months [26, 27, 29–31, 33, 34, 42]. The median follow-up time was 9 months. Median study sample size was 260 ranging from 104 to 6963 participants.

**Table 1:** Characteristics of included studies

Authors (year), Country	Design	Sample Size	Intervention	Study Population Characteristics	Study aim
Adler D. (2004), USA [42]	Randomized with 18-months follow-up	533	Medication review, pharmaceutical meeting and recommendations to physicians	Patients met DSM-IV criteria for major depressive disorder and/or dysthymia	To examine the clinical pharmacist’s role in the treatment of depression in primary care
Carter B. (2009), USA [25]	Cluster-randomized with 6-months follow-up	302	Medication review, pharmaceutical meeting and recommendations to physicians Measures taking and demand of biological tests	Patients over 21 years of age having a diagnosis of essential hypertension taking 0 to 3 antihypertensive medications without diabetes mellitus	To evaluate if a physician and pharmacist collaborative model in community-based medical offices could improve BP control
Carter B. (2015), USA [26]	Cluster-randomized with 24-months follow-up	625	Medication review, pharmaceutical meeting and recommendations to physicians	Patients with no BP control	To evaluate the pharmacist-physicians collaboration could improve BP control
Carter B. (2018), USA [27]	Cluster-randomized with 12-months follow-up	302	Medication review, pharmaceutical meeting and recommendations to physicians	Patients over 50 years with a history of at least one of the following: diabetes mellitus, hypertension, hypercholesterolemia	To assess whether the pharmacist intervention would be successfully implemented into private family physician offices
Chen Z. (2013), USA [28]	Cluster-randomized with 6-months follow-up	374	Medication review, pharmaceutical meeting and recommendations to physicians	Patients aged 21 to 85 years and were receiving treatment with 0 to 3 antihypertensive agents with no changes to their regimen within the past 4 weeks	To detail the changes in specific antihypertensives associated with the differences in 24-hour BP following a physician-pharmacist co-management
Finley P. (2002), USA [39]	Randomized with 6-months follow-up	220	Medication review, pharmaceutical meeting and recommendations to physicians	Patients suffering from depression and subsequently received prescriptions for antidepressant medication	To evaluate the impact of a collaborative pharmacy practice model on the treatment of depression in primary care
Finley P. (2003), USA [40]	Randomized with 6-months follow-up	125	Medication review, pharmaceutical meeting and recommendations to physicians	Patients who need antidepressant medication	To test the effects of this collaborative care model on drug adherence rates, patient outcomes, provider and patient satisfaction, and medical resource utilization
Heisler M. (2012), USA [29]	Cluster-randomized with 14-months follow-up	4100	Medication review, pharmaceutical meeting and recommendations to physicians Measure of BP and demand of biological tests	Patients with diabetes mellitus had persistent poor BP control and poor refill adherence or insufficient medication intensification	To evaluate if the pharmacist intervention improve BP control
Hogg W. (2009), Canada [30]	Randomized with 18-months follow-up	241	Medication review, pharmaceutical meeting and recommendations to physicians	Patients over 50 years, rostered in the practice, and considered by their family physicians to be good candidates to benefit from additional medical resources and at risk of functional decline, physical deterioration, or experiencing an event requiring emergency services	To evaluate the benefits of home-based multidisciplinary team management involving a nurse practitioner, a pharmacist, and a general practitioner working collaboratively on providing care to community-dwelling patients who were at risk of poor health outcomes
Jameson J. (2010), USA [31]	Randomized with 12-months follow-up	104	Medication review, pharmaceutical meeting and recommendations to physicians	Patients having HbA1c levels of 9.0% or higher or non-office visits within 12 months	To investigate the effect of pharmacist management of poorly controlled diabetes mellitus in a community-based primary care group
Lenaghan E. (2007), UK [41]	Randomized with 6-months follow-up	136	Medication review, pharmaceutical meeting and recommendations to physicians	Patients over 80 years, living in their own homes, who were prescribed at least four oral daily medicines	To study whether a home-based intervention in an at-risk elderly population could reduce hospital admissions
Omran D. (2015), Canada [32]	Randomized with 6-months follow-up	260	Medication review, pharmaceutical meeting and recommendations to physicians	Patients with type 2 diabetes	To determine whether observed improvements in BP resulted from pharmacists’ recommendations to improve antihypertensive medication management or patients’ adherence to antihypertensive medications
Pape G. (2011), USA [33]	Cluster-randomized with 24-months follow-up	6963	Medication review, pharmaceutical meeting and recommendations to physicians	Patients with type 2 diabetes	To evaluate the impact of remote physician-pharmacist team-based care on cholesterol levels in patients with diabetes mellitus
Sellors J. (2003), Canada [43]	Randomized with 5-months follow-up	889	Medication review, pharmaceutical meeting and recommendations to physicians	Patients aged 65 years or more, taking 5 medications	To evaluate pharmacist intervention could reduce daily units of medication taken and improving patient outcomes
Simpson S. (2011), Canada [34]	Randomized with 12-months follow-up	260	Medication review, pharmaceutical meeting and recommendations to physicians Measures taking and demand of biological tests	Patients with type 2 diabetes	To study the effect of adding pharmacists to extant multidisciplinary primary care teams on cardiovascular risk-factor management in type 2 diabetes
Smith S. (2016), USA [35]	Cluster-randomized with 9-months follow-up	169	Medication review, pharmaceutical meeting and recommendations to physicians	Patients without BP control and taking 3 or more antihypertensive medications	To compare a physician-pharmacist collaborative care model to usual hypertension care
Tahaineh L. (2011), Jordan [36]	Randomized with 6-months follow-up	159	Medication review, pharmaceutical meeting and recommendations to physicians	Patients with dyslipidemia	To evaluate the impact of implementing a clinical pharmacy service on achieving lipid profile goals in primary care setting
Tobari H. (2010), Japan [37]	Randomized with 6-months follow-up	132	Medication review, pharmaceutical meeting and recommendations to physicians	Patients 40–79 years of age with hypertension	To evaluate physician-pharmacist cooperation can reduce antihypertensive medication use and cardiovascular risk factors in patient with mild to moderate hypertension by improving BP control
Weber C. (2010), USA [38]	Cluster-randomized with 9-months follow-up	179	Medication review, pharmaceutical meeting and recommendations to physicians	Patients aged 21 to 85 years and were receiving treatment with 0 to 3 antihypertensive agents with no changes to their regimen within the past 4 weeks	To report the results of 24-hour ambulatory BP monitoring obtained during a pharmacist-physician collaborative model of hypertension management

Abbreviations: BP, blood pressure; HbA1c, glycosylated hemoglobin

### Characteristics of pharmacist intervention

Supplementary material 2 lists the characteristics of pharmacist intervention. Most studies investigated a collaborative intervention involving only a physician and a pharmacist [25–29, 32, 33, 35–38, 41–43]. Two studies involved a physician, a pharmacist, and a nurse [30, 31]. Three studies involved a pharmacist and an allied health team consisting of physicians, nurses, dietitians, psychotherapists, care managers, social workers or psychiatrists [34, 39, 40].

Overall, the pharmacist intervention typically involved medication review, patient interviews, and recommendations to physicians. The medication review was performed using data recorded in an electronic medical record database, then completed with a telephone or face-to-face patient interview [26, 34–36, 38, 39, 43]. One study involved nurses who developed an individualized care plan with the patient and in consultation with the pharmacist and the physician [30]. The frequency of clinical contact with patients varied from 2 [41, 43] to 12 or more contacts [27]. The physician was free to accept or reject pharmacist recommendations except in one study where the pharmacist had the authorization to directly modify medications, and the physician was solely informed [29]. In all the other studies, recommendations to physicians were provided either during face-to-face contact [25, 26, 28, 37, 41–43], or by telephone [37, 40, 42] or email [26, 27, 29, 33, 35, 39, 42, 43]. The acceptance rate for pharmacist interventions was reported in seven studies and was between 76.6% and 96.2% [25, 27, 28, 33, 36, 38, 43].

However, three studies, also assessed additional activities combined with the typical pharmacist intervention. Heisler et al. (2012), investigated an intervention in which the pharmacist suggested and prescribed treatment changes directly to the patient [29]. Three studies investigated a pharmacist intervention that involved taking clinical measurements (blood pressure, heart rate or weight) and laboratory tests [25, 29, 34].

In all studies, participants in the control group did not benefit from pharmacist intervention; instead, they were exclusively received usual care from physicians alone [25–29, 31, 33, 35–38, 40, 41, 43] or from primary care teams composed on physicians and nurses [30, 32, 34, 39, 42].

### Effect of pharmacist intervention on patient-related outcomes

**Fig. 2:**
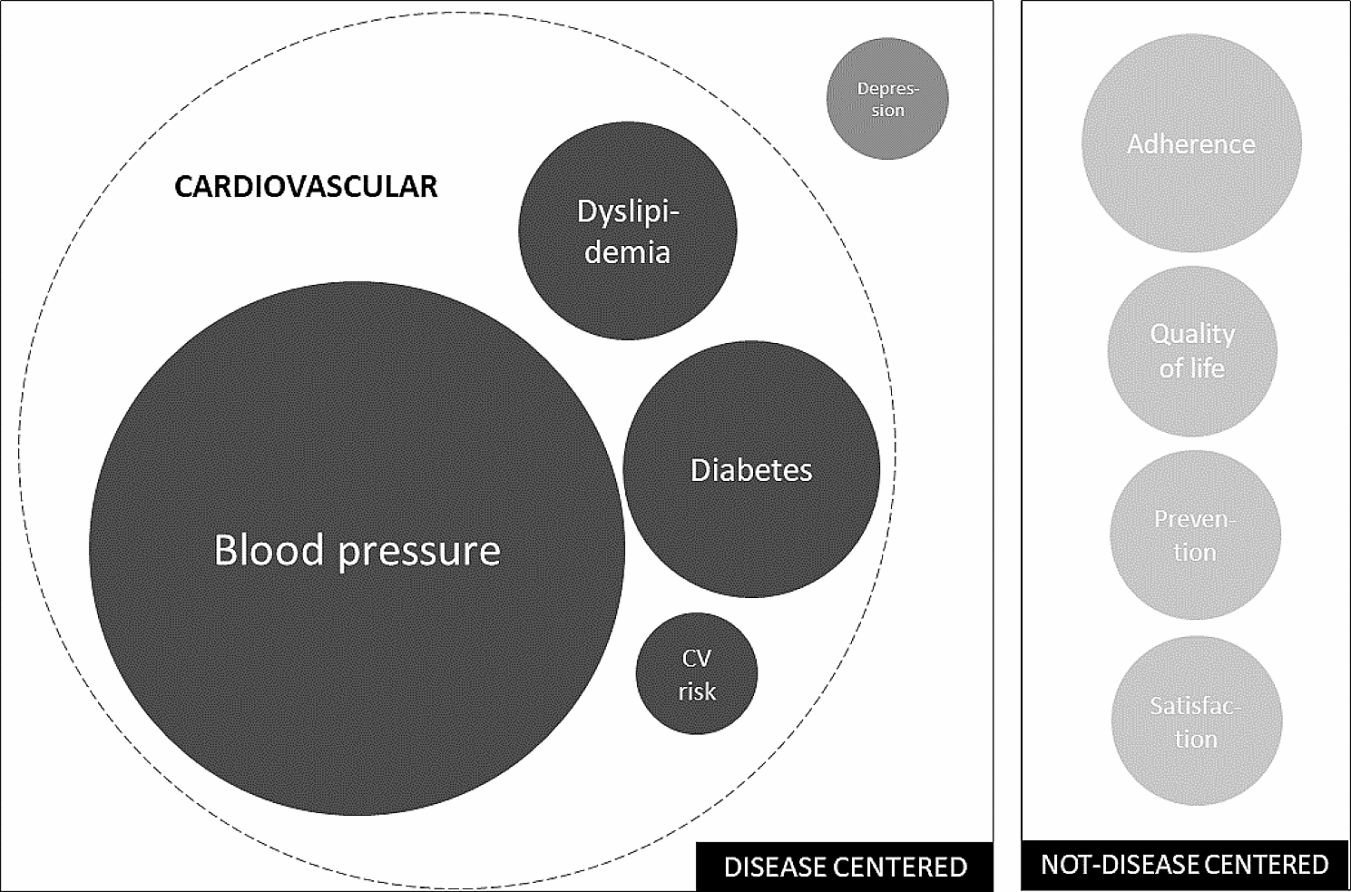
Illustration of Investigated Topic Frequencies in Selected Studies. Legend: the bigger the circle, the more studied the topic. Abbreviations: CV risk: cardiovascular risk

Figure 2 illustrates the various conditions in which the pharmacist intervention was investigated. Of the 19 studies included, 13 involved patients with cardiovascular risk factors (hypertension [25–29, 35, 37, 38], diabetes [27, 29, 31–34] and/or dyslipidemia [27, 36]). Three studies included patients with depression [39, 40, 42] and three studies included elderly people with multiple chronic diseases [30, 41, 43].

Table 2 lists the outcome measures and significant results studied for the various pathologies. We found that the effect of pharmacist intervention varied depending on the outcome measure and follow-up duration. In supplementary material 3, a table lists the outcome measures and significant and non-significant results.

**Table 2:** Summary of outcomes with significant results

	Outcome criteria	Intervention vs. control group	Difference in change between Intervention and Control group	P value
Carter B. (2009) [25]	**Proportion of patients with BP control at 6 months (%)**	**63.9 vs. 29.9 (OR 3.2, 95% CI 2.0-5.1)**	**-**	*p* < 0.001
	Proportion of patients with BP control without diabetes mellitus at 6 months (%)	68.8 vs. 32.4 (OR 3.9, 95% CI 3.1-5.0)	-	*p* < 0.001
	Proportion of patients with BP control with diabetes mellitus at 6 months (%)	45.5 vs. 26.1 (OR 4.7, 95% CI 1.7–13.1)	-	*p* = 0.003
	**SBP at 6 months (mmHg)**	**-20.7 vs. -6.8**	**-**	*p* < 0.05
Carter B. (2015) [26]	SBP at 9 months (mmHg)	131.6 vs. 138.2	-6.1 (95% CI-9.75 to -2.39) *	*p* = 0.002
	DBP at 9 months (mmHg)	76.3 vs. 78.0	-2.9 (95% CI -4.85 to -0.93) *	*p* = 0.005
	SBP in minority ethnicity subjects at 9 months (mmHg)	133.0 vs. 140.3	-6.4 (95% CI -11.16 to -1.68) *	*p* = 0.009
	DBP in minority ethnicity subjects at 9 months (mmHg)	77.9 vs. 78.8	-2.9 (95% CI -5.88 to -0.08) *	*p* = 0.044
Carter B. (2018) [27]	Proportion of patients with Body Mass Index screening and follow-up at 12 months (%)	68.0 vs. 37.4	**-**	*p* < 0.001
	Proportion of patients with alcohol use screening at 12 months (%)	98.0 vs. 88.2	-	*p* < 0.001
Chen Z. (2013) [28]	**24-hour SBP at 6 months (mmHg)**	**120.4 vs. 131.8**	-	*p* < 0.001
	Proportion of patients with BP control at 6 months (%)	75.6 vs. 50.0	-	*p* < 0.001
Finley P. (2002) [39]	**6-months Medication Possession ratio (MPR)**	**0.811 vs. 0.659**	**-**	*p* < 0.005
	Patient satisfaction survey results at 6 months	Patients in intervention group were more likely to have received an antidepressant previously: 25.0% vs. 19.0%	-	*p* = 0.044
Finley P. (2003) [40]	Patient satisfaction survey results at 6 months	Patients in the intervention group expressed greater satisfaction than did control group with the personal nature of care, availability of providers, ability of providers to listen, explanation of why antidepressants were prescribed, explanation of how to take the antidepressants and patient’s overall satisfaction with the health maintenance organization	-	*p* < 0.05
Hogg W. (2009) [30]	Number of patients with influenza vaccination	-	0.087 (95% CI 0.012–0.162) **	*p* = 0.023
	Number of patients with screening for colorectal cancer	-	0.167 (95% CI 0.046–0.288) **	*p* = 0.0070
	Number of patients with hearing examination	-	0.273 (95% CI 0.106–0.44) **	*p* = 0.0016
	Number of patients with eye examination	-	0.220 (95% CI 0.076–0.364) **	*p* = 0.0029
Jameson J. (2010) [31]	HbA1c for male patients at 12 months (%)	-1.90 vs. -0.15	-	*p* = 0.03
	Proportion of patients who achieved at least a 1.0% decrease in HbA1c at 12 months (%)	67.3 vs. 41.2	-	*p* = 0.02
	Proportion of patients who achieved at least a 1.0% decrease in HbA1c for patients of nonwhite race/ethnicity at 12 months (%)	56.3 vs. 22.7	-	*p* = 0.03
	Proportion of patients who achieved at least a 1.0% decrease in HbA1c for male patients at 12 months (%)	72.0 vs. 28.0	-	*p* = 0.002
Pape G. (2011) [33]	**LDL-C at 24 months (mg/dl)**	**83.0 vs. 95.0**	-	*p* < 0.001
	**Proportion of patients with LDL-C at target goal at 24 months (%)**	**78.0 vs. 50.0**	-	*p* = 0.003
	**Proportion of patients with LDL-C at target if they were not at goal baseline at 24 months (%)**	**74.0 vs. 48.0**	-	*p* = 0.001
	Proportion of patients with LDL-C test within the past 12 months at 24 months (%)	95.0 vs. 82.0	-	*p* = 0.04
Simpson S. (2011) [34]	**Proportion of patients with diminution of 10.0% of SBP at 12 months (%)**	**37.0 vs. 23.0 (OR 1.91, 95% CI 1.11–3.28)**	**-**	*p* = 0.02
	**Proportion of patients with elevated blood pressure at baseline with diminution of 10.0% of SBP at 12 months (%)**	**50.0 vs. 28.0 (OR 2.55, 95% CI 1.30–5.01)**	**-**	*p* = 0.007
	**SBP at 12 months (mmHg)**	-7.4 vs. -2.5	-4.9 (95% CI -8.7 to -1.0) *	*p* = 0.002
	**DBP at 12 months (mmHg)**	-2.3 vs. 0.6	-2.9 (95% CI -5.6 to -0.2) *	*p* < 0.05
	**SBP for patients with elevated blood pressure at 12 months (mmHg)**	**-13.9 vs. -6.7**	-	*p* = 0.002
	Predicted 10-year risk of cardiovascular events at 12 months (%)	-2.7 vs. -1.2	-1.5 (95% CI -0.2 to 3.3) *	*p* = 0.005
Smith S. (2016) [35]	SBP at 9 months (mmHg)	132 ± 16 vs. 141 ± 20	-6.62 (95% CI -12.8 to -0.44) *	*p* = 0.036
	Proportion of patients with an improvement from low to high medication adherence minority ethnicity subjects (antihypertensive use) at 9 months (%)	8.1 vs. 0.0	-	*p* = 0.016
Tahaineh L. (2011) [36]	**Proportion of patients reached their LDL-C goal at 6 months (%)**	94.5 vs. 71.2	-	*p* < 0.001
	Proportion of patients reached their total cholesterol goal at 6 months (%)	87.7 vs. 73.1	-	*p* = 0.038
	Proportion of patients reached their HDL-C goal at 6 months (%)	28.8 vs. 50.0	-	*p* = 0.016
Tobari H. (2010) [37]	DBP at home at 6 months (mmHg)	-3.3 (-4.8 to -1.8) vs. -1.4 (-2.9 to 0.1)	-2.8 (95% CI -5.5 to -0.1) *	*p* = 0.04
	Body Mass Index at 6 months (kg/m²)	-0.4 (-0.7 to -0.2) vs. 0.0 (-0.2 to 0.2)	-0.4 (95% CI -0.7 to -0.1) *	*p* = 0.008
	Sodium reduction score at 6 months	1.3 (0.9 to 1.7) vs. 0.0 (-0.4 to 0.4)	1.2 (95% CI 0.5-2.0) **	*p* = 0.002
	Number of smokers at 6 months	9.0 [14.0] vs. 19.0 [30.0]	0.4 (95% CI 0.2–0.9) **	*p* = 0.04
Weber C. (2010) [38]	**Change in 24-hour SBP at 9 months (mmHg)**	**136.0 to 130.5 vs. 135.5 to 121.4**	**-**	*p* < 0.001
	**Change in 24-hour DBP at 9 months (mmHg)**	**76.6 to 73.7 vs. 76.0 to 69.2**	**-**	*p* < 0.001

Abbreviations: BP, blood pressure; DBP, diastolic blood pressure; HbA1c, glycosylated hemoglobin; HDL-C, high-density lipoprotein cholesterol; LDL-C, low-density lipoprotein cholesterol; SBP, systolic blood pressure

Legend: **Bold text**:primary outcomes of study;^*^Negative values indicate Intervention Group has larger change; ^**^Positive values indicate Intervention Group has larger change

Concerning blood pressure reduction, most studies reported that the pharmacist intervention significantly lowered blood pressure [25, 26, 28, 34, 35, 37, 38] with a follow-up duration ranging from 6 to 12 months. Four studies did not report any effect [27, 29, 30, 33]. Concerning blood pressure control, defined as blood pressure measured lower than 130/80 mmHg for patients with diabetes mellitus or chronic disease and lower than 140/90 for all other patients, two studies found pharmacist intervention had a positive effect [25, 28] and four studies did not find any effect [26, 27, 35, 37].

Seven studies measured glycosylated hemoglobin (HbA1c) levels [29, 31–36]. However, only one study reported finding significantly improved HbA1c levels following a pharmacist intervention, among males and ethnic minority subgroups [31].

Concerning pharmacist interventions to reduce cardiovascular diseases and risk factors, Simpson et al. (2011) reported a significant reduction of 1.5% (95% CI -0.2 to 3.3) in predicted 10-year cardiovascular event risk [34]. More specifically, Tobari et al. (2010) reported lower Body Mass Index (BMI), sodium score and fewer smokers, but no effect was demonstrated among patients with alcohol consumption > 23 g/day or with brisk walking > 30 min/day [37].

Furthermore, among those interventions to control dyslipidemia [27, 29, 33, 34, 36], two studies reported that pharmacist intervention resulted in significantly lower low-density lipoprotein cholesterol (LDL-C) after 6 or 24 months [33, 36] and total cholesterol after 6 months [36] but three studies did not demonstrate any effect [27, 29, 34].

Two studies focused on depressive disorders with one showing that patients expressed greater satisfaction than controls with a pharmacist intervention but neither reported an effect of pharmacist intervention on patient related outcome [40, 42].

Five studies evaluated medication adherence [25, 32, 35, 39, 40], two of which reported a positive effect of pharmacist intervention on patient adherence to medication, including a *post-hoc* analysis among an ethnic minority group [35, 39]. Three studies did not report significant results [25, 32, 40].

None of the three studies identified demonstrated an effect of pharmacist intervention on the quality of life in older people [30, 41, 43].

For the studies evaluating the pharmacist role in preventative medicine, a positive effect was observed for influenza vaccination, colorectal cancer screening, hearing and eye examination [30], BMI screening and follow-up and alcohol use [27]. However, no effect was observed on breast and cervical cancer screening [30], diabetic foot examination, dilated eye examination, microalbumin measurement and proportion of smokers advised to quit [27]. Pape et al. (2011) found that a higher proportion of patients underwent a LDL-C test in the intervention group [33].

Lastly, a positive effect of pharmacist intervention was observed in an interprofessional collaboration. Patients appreciated the personal nature of care, provider availability, provider ability to listen, explanation of why antidepressants were prescribed, and the explanation of how to take the antidepressants and, overall, patients were satisfied with the pharmacist intervention [39, 40]. Pape et al. (2011) did not find significant results [33].

**Fig. 3:**
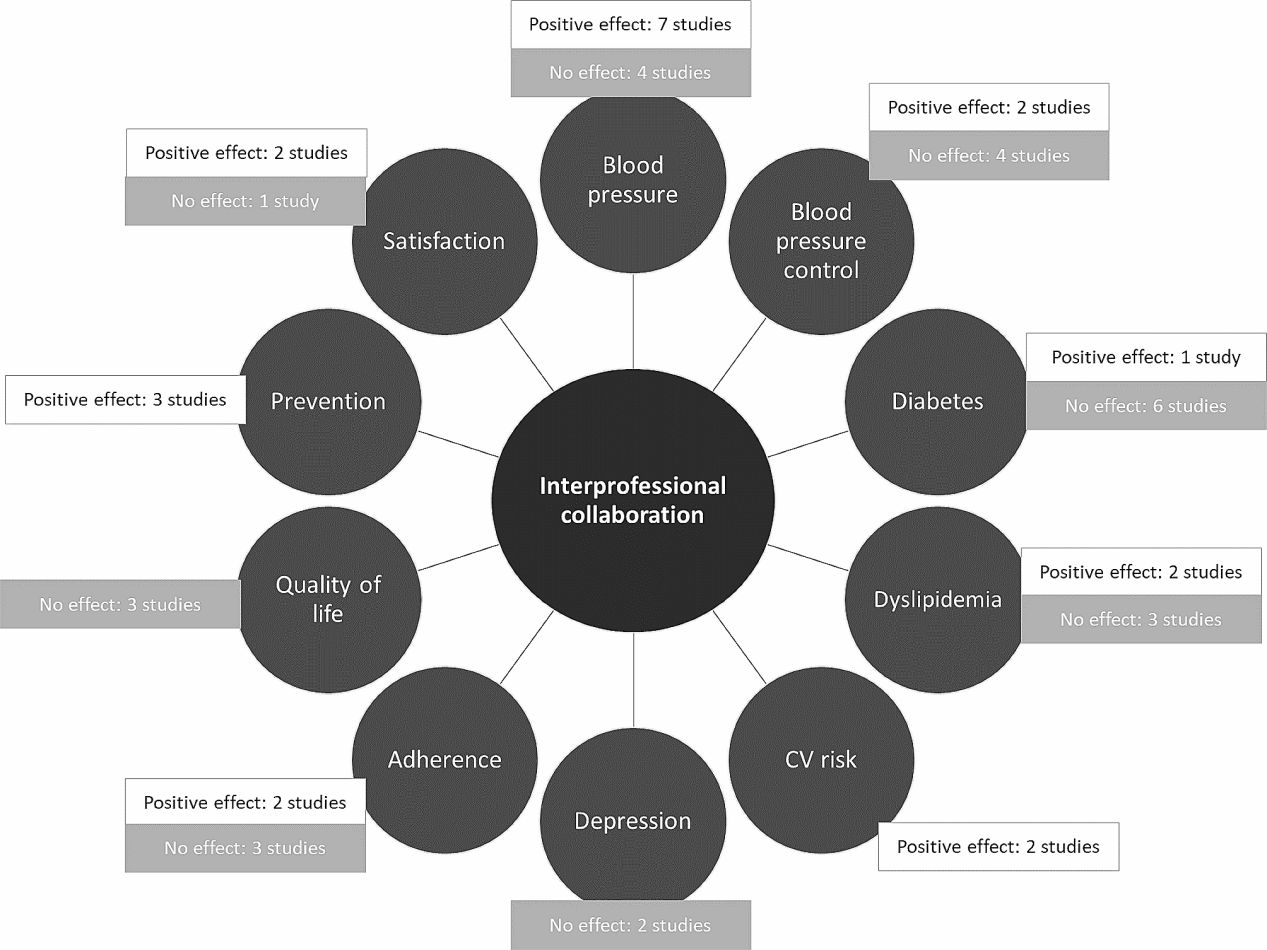
: Synthesis of the impact of interprofessional collaboration categorized by subject. Abbreviations: CV risk: cardiovascular risk

Fig. 3 provides a synthesized overview of the evidence pertaining on interprofessional collaboration

### Critical appraisal of studies

According to the ICROMS quality assessment, six studies had a low risk of bias [30, 31, 33, 34, 37, 41], meeting both minimum score and mandatory criteria​. Nine studies had a moderate risk of bias; minimum ICROMS scores were met but mandatory scores were not [25–29, 35, 38, 40, 43]. The remaining four studies had a high risk of bias, with neither minimum or mandatory scores being met [32, 36, 39, 42]. The results are summarized in Table 3.

**Table 3:** Risk of bias assessment using ICROMS

Study	Dimension							Total score	Minimum score met (≥ 22)	Mandatory criteria met
	(1) Clear aims and justification	(2) Managing bias in sampling and between groups	(3) Managing bias in outcome measurement and blinding	(4) Managing bias in flow-up	(5) Managing bias in other study aspects	(6) Analytical rigour	(7) Managing bias in reporting/ethical considerations			
Adler D. (2004) [42]	2	3	2	1	2	2	6	18	No	No
Carter B. (2009) [25]	2	3	6	4	2	2	9	28	Yes	No
Carter B. (2015) [26]	2	2	4	2	2	2	9	23	Yes	No
Carter B. (2018) [27]	2	4	3	4	2	2	9	26	Yes	No
Chen Z. (2013) [28]	2	3	6	2	2	2	8	25	Yes	No
Finley P. (2002) [39]	2	0	4	0	2	2	5	15	No	No
Finley P. (2003) [40]	2	4	5	0	2	2	7	22	Yes	No
Heisler M. (2012) [29]	2	4	4	5	2	2	7	26	Yes	No
Hogg W. (2009) [30]	2	4	6	0	2	2	7	23	Yes	Yes
Jameson J. (2010) [31]	2	4	6	5	2	2	8	29	Yes	Yes
Lenaghan E. (2007) [41]	2	4	6	6	2	0	6	26	Yes	Yes
Omran D. (2015) [32]	2	4	5	0	2	1	7	21	No	Yes
Pape G. (2011) [33]	2	4	6	0	2	2	8	24	Yes	Yes
Sellors J. (2003) [43]	2	4	4	4	2	2	7	25	Yes	No
Simpson S. (2011) [34]	2	4	6	6	2	2	9	31	Yes	Yes
Smith S. (2016) [35]	2	2	4	2	2	2	8	22	Yes	No
Tahaineh L. (2011) [36]	2	2	3	0	1	2	8	18	No	No
Tobari H. (2010) [37]	2	4	5	5	2	2	8	28	Yes	Yes
Weber C. (2010) [38]	2	2	6	6	2	2	7	27	Yes	No

## Discussion

This systematic review identified 19 randomized controlled trials evaluating the effects of pharmacist interventions in primary care conducted in various countries and different health care settings. Most studies concerned a physician-pharmacist collaboration and only five studies included a physician and other health professionals. All studies included a standard pharmacist intervention involving medication review, patient interview, and recommendations to the physician. However, three studies also included additional responsibilities. Interestingly we found an over-representation of RCTs among patients with cardiovascular risk factors such as hypertension, diabetes, or dyslipidemia. Only six studies evaluated pharmacist involvement in interprofessional collaboration among patients with other pathologies and with mixed results.

The standard pharmacist intervention involving medication review, patient interview, and recommendations to the physician is in accordance with previously described literature [44, 45]. However, some studies also included additional responsibilities in the intervention including new medicine prescription, laboratory assessments or deprescribing which is also consistent with existing literature [44, 46]. The additional responsibilities identified in our review should be compared with the roles assigned to community pharmacists in different countries. Notably, the implementation of legislation supporting pharmacist prescribing in the United Kingdom, Canada, the United States, and New Zealand may explain the emergence of these new responsibilities highlighted in our review [47, 48]. In this collaboration, physicians were also core members. Previous authors have analyzed pharmacist-physician collaboration [45, 49, 50], either to determine factors influencing collaboration [51] or to explore attitudes towards interprofessional collaboration [52]. Only five studies involved other health professionals, which was less than expected.

Because of the limited number of studies incorporating more than two professionals, we were unable to investigate the correlation between the number of professions in a collaboration and its impact on patient health and satisfaction outcomes. However, this variable was explored in a recent systematic review which found no association between the number of professions in the interprofessional collaboration and HbA1c reduction [53].

A large majority of included articles evaluated the role of a pharmacist intervention in managing cardiovascular diseases and risk factors, particularly blood pressure. This supports evidence from recent reviews [44, 53, 54]. Indeed, the meta-analysis of Tan et al. (2014), including four studies that were also included in our review [29, 30, 34, 37], reported that interventions involving pharmacists or nurses were associated with significantly improved BP control [44]. Moreover, some included studies reported that pharmacist care improved lipid parameters, notably LDL-C levels, as well as increasing the proportion of patients who achieved targeted levels which is consistent with existing literature [55]. In contrast, results concerning HbA1c were conflicting. Whilst we found only one study reporting positive effects, previous research reported more studies with positive effects on HbA1c with pharmacist interventions [44, 56, 57]. Tan et al. (2014) found four studies with positive effects and two studies with no effect and concluded with a meta-analysis that HbA1c reduced by 0.88% in the intervention group [44]. In Pousinho et al. (2016) review, HbA1c was considered as an outcome measure in 26 studies and 24 studies reported a greater improvement in this outcome in the intervention group compared with the control group [56]. The divergence in results could be explained by the fact that in our included studies, the pharmacist interventions were not directly focused on diabetes but were a secondary outcome measure.

Concerning quality of life and patient satisfaction, our review did not report significant results. Many different assessment tools were used to explore these two variables which may explain these results. Similarly, an umbrella review conducted by Abdulrhim et al. (2020) was unable to conclude about improvements in quality of life for patients with diabetes due to the use of diverse quality of life assessment tools [57].

### Bias of included studies

Only randomized controlled trials were included in this systematic review because they provide the most reliable evidence on intervention effectiveness. However, the quality assessment revealed that the majority of the included studies had a moderate risk of bias mainly due to their methodological quality. No article had a methodology with double blinding, which is a strong criterion in quality assessment. This absence was anticipated, considering the nature of the interventions, where professionals involvement precludes blinding. Therefore, the certainty of evidence supporting the conclusions about intervention effectiveness is low.

### Limitations

Despite following PRISMA guidelines, our systematic review did have some limitations. Firstly, the exclusive focus on RCTs improves the robustness of the review. However, there is limited availability of relevant RCTs for significant primary care topics, such as chronic obstructive pulmonary disease [58], or infection [59], which were consequently omitted from our synthesis. Secondly, even though we chose to only include RCTs, it was still difficult to group the outcomes to assess the effect of pharmacist involvement in a primary care interprofessional team. A number of factors explain this, including the differences in patient populations, follow-up durations, instruments used to measure outcome and intervention complexity. These discrepancies deserve further thought and investigation into appropriate core outcome measure to be used in primary care research and strategies and methods currently used to optimize implementation of complex interventions [60, 61]. Thirdly, the quality assessment revealed that the majority of included studies had a moderate risk of bias due to their methodological quality. Finally, there is no existing consensus defining interprofessional collaboration [62, 63] and the search strategy had to consider several terms. We used Mesh terms in the search strategy to be more specific. Moreover, to our knowledge, there are no recommendations about professions which should be involved in a primary care interprofessional team, and this made it difficult to select studies.

## Conclusion

This review revealed that pharmacists are mainly responsible for medication review, interview with patients and recommendations to physicians, and most commonly collaborate with physicians. Pharmacist collaboration particularly improved blood pressure and cholesterol control. Our review highlights the need for further controlled studies into interprofessional collaboration interventions involving a pharmacist in primary care across the full range of medical conditions.

### Electronic supplementary material

Below is the link to the electronic supplementary material.

Supplementary Material 1

Supplementary Material 2

Supplementary Material 3

## Data Availability

The dataset supporting the conclusions of this article is included within the article and its supplementary materials.

## Abbreviations

BMI: Body Index Mass
BP: blood pressure
DBP: diastolic blood pressure
HbA1c: glycosylated hemoglobin
HDL-C: high-density lipoprotein cholesterol
LDL-C: low-density lipoprotein cholesterol
RCT: randomized controlled trials
SBP: systolic blood pressure
