# Supplementary material for: Involving community pharmacists in interprofessional collaboration in primary care: a systematic review
Source: BMC Prim Care. 2024 Apr 1;25:103. doi: 10.1186/s12875-024-02326-3 (PMC10983710; doi:10.1186/s12875-024-02326-3)
Supplement: Supplementary file 1 — Supplementary Material 1 [file 12875_2024_2326_MOESM1_ESM.docx]

*Additional file 1: Information regarding the search strategy*

“The following search strategy was used in PubMed (from inception to 1^st^ July 2021):

*("Intersectoral Collaboration"[Mesh] OR "Cooperative Behavior"[Mesh] OR "Patient Care Team"[Mesh:NoExp]) AND ("Primary Health Care"[Mesh]) AND ("Outcome and Process Assessment, Health Care"[Mesh]).*

- 1977 records identified.

The following search strategy was used in PubMed (from 1^st^ July 2021 to 1^st^ November 2022):

*("Intersectoral Collaboration"[Mesh] OR "Cooperative Behavior"[Mesh] OR "Patient Care Team"[Mesh:NoExp]) AND ("Primary Health Care"[Mesh]) AND ("Outcome and Process Assessment, Health Care"[Mesh]).*

- 6 records identified.

The following search strategy was used in Embase, PsychINFO and CINAHL (from inception to 1^st^ July 2021):

*“intersectoral collaboration”, “cooperative behavior”, “patient care team” AND “primary health care” AND “outcome and process assessment, health care.”*

- 1178 records identified in Embase, 193 in PsycINFO and 146 in CINAHL.”
