# Supplementary material for: Involving community pharmacists in interprofessional collaboration in primary care: a systematic review
Source: BMC Prim Care. 2024 Apr 1;25:103. doi: 10.1186/s12875-024-02326-3 (PMC10983710; doi:10.1186/s12875-024-02326-3)
Supplement: Supplementary file 2 — Supplementary Material 2 [file 12875_2024_2326_MOESM2_ESM.docx]

*Additional file 2: Characteristics of pharmacist intervention*

| **Authors (year), Country** | **Professions involved** | **Comparator** | **Supports used for medication review** | **Pharmaceutical meetings with the patient** | **Communication with the physician** | **Acceptance rate of recommendations by physicians** |
| --- | --- | --- | --- | --- | --- | --- |
| Adler D. (2004), USA (44) | Pharmacist and Physician | Usual care by PCT | Electronic database | minimum 9 times | Face-to-face, phone and electronic mail | NS |
| Carter B. (2009), USA (27) | Pharmacist and Physician | Usual care by the physician | Electronic database | minimum 3 times | Face-to-face | 96.2% |
| Carter B. (2015), USA (28) | Pharmacist and Physician | Usual care by the physician | Electronic database and pharmaceutical meeting | minimum 6 times | Face-to-face and electronic mail | NS |
| Carter B. (2018), USA (29) | Pharmacist and Physician | Usual care by the physician | Electronic database | Every 1 to 2 weeks if there were problems and every 1 to 2 months as problems resolved | Electronic mail | 88.8% |
| Chen Z. (2013), USA (30) | Pharmacist and Physician | Usual care by the physician | Electronic database | minimum 3 times | Face-to-face | 95.0% |
| Finley P. (2002), USA (41) | Pharmacist, Physician, Care manager and Psychiatric | Usual care by PCT | Electronic database and pharmaceutical meeting | 5 times | Electronic mail | NS |
| Finley P. (2003), USA (42) | Pharmacist, Physician, Care manager and Psychiatric | Usual care by the physician | Electronic database and meeting by a care manager | 5 times | Phone and electronic mail | NS |
| Heisler M. (2012), USA (31) | Pharmacist and Physician | Usual care by the physician | Electronic database | 5 times | Electronic mail | NS |
| Hogg W. (2009), Canada (32) | Pharmacist, Physician and Nurse | Usual care by PCT | Electronic database | NS | NS | NS |
| Jameson J. (2010), USA (33) | Pharmacist, Physician and Nurse | Usual care by the physician | Electronic database | As required | NS | NS |
| Lenaghan E. (2007), UK (43) | Pharmacist and Physician | Usual care by the physician | Electronic database and pharmaceutical meeting | 2 times | Face-to-face | NS |
| Omran D. (2015), Canada (34) | Pharmacist and Physician | Usual care by PCT | Electronic database | NS | NS | NS |
| Pape G. (2011), USA (35) | Pharmacist and Physician | Usual care by the physician | Electronic database | NS | Electronic mail | 93.0% |
| Sellors J. (2008), Canada (45) | Pharmacist and Physician | Usual care by the physician | Electronic database and pharmaceutical meeting | 2 times | Face-to-face and electronic mail | 76.6% |
| Simpson S. (2011), Canada (36) | Pharmacist, Physician, Nurse, Dietitian, Psychotherapist and social worker | Usual care by PCT | Electronic database and pharmaceutical meeting | NS | NS | NS |
| Smith S. (2016), USA (37) | Pharmacist and Physician | Usual care by the physician | Electronic database and pharmaceutical meeting | Minimum 5 times | Electronic mail | NS |
| Tahaineh L. (2011), Jordan (38) | Pharmacist and Physician | Usual care by the physician | Electronic database and pharmaceutical meeting | 3 times | NS | 90.3% |
| Tobari H. (2010), Japan (39) | Pharmacist and Physician | Usual care by the physician | Electronic database | Minimum 4 times | Face-to-face and phone | NS |
| Weber C. (2010), USA (40) | Pharmacist and Physician | Usual care by the physician | Electronic database and pharmaceutical meeting | 5 times | NS | 95.9% |

Abbreviations: NS, not specified ; PCT, primary care team
