# Supplementary material for: Involving community pharmacists in interprofessional collaboration in primary care: a systematic review
Source: BMC Prim Care. 2024 Apr 1;25:103. doi: 10.1186/s12875-024-02326-3 (PMC10983710; doi:10.1186/s12875-024-02326-3)
Supplement: Supplementary file 3 — Supplementary Material 3 [file 12875_2024_2326_MOESM3_ESM.docx]

*Additional file 3: Summary of outcomes with significant results and non-significant results*

|  | **Outcome criteria** | **Intervention vs control group** | **Difference in change between Intervention and Control group** | ***P* value** |
| --- | --- | --- | --- | --- |
| Adler D. (2004), USA (44) | **Depression outcomes (mBDI score) at 3 months** | **NS** | **NS** |  |
|  | **Depression outcomes (mBDI score) at 6 months** | **NS** | **NS** |  |
|  | Depression outcomes (mBDI score) for patients not on antidepressant at enrollment at 3 months | NS | NS |  |
|  | Depression outcomes (mBDI score) for patients not on antidepressant at enrollment at 6 months | NS | NS |  |
| Carter B. (2009), USA (27) | **Proportion of patients with BP control at 6 months (%)** | **63.9 vs 29.9 (OR 3.2, 95% CI 2.0-5.1)** | **-** | p < 0.001 |
|  | Proportion of patients with BP control without diabetes mellitus at 6 months (%) | 68.8 vs 32.4 (OR 3.9, 95% CI 3.1-5.0) | - | p < 0.001 |
|  | Proportion of patients with BP control with diabetes mellitus at 6 months (%) | 45.5 vs 26.1 (OR 4.7, 95% CI 1.7-13.1) | - | p = 0.003 |
|  | **SBP at 6 months (mmHg)** | **-20.7 vs -6.8** | **-** | p < 0.05 |
|  | **DBP at 6 months (mmHg)** | **NS** | **NS** |  |
|  | Proportion of patients with poor self-reported medication adherence at 6 months (%) | **NS** | **NS** |  |
| Carter B. (2015), USA (28) | **Proportion of patients with BP control at 9 months (%)** | **NS** | **NS** |  |
|  | **Proportion of patients with BP control in minority ethnicity subjects at 9 months (%)** | **NS** | **NS** |  |
|  | SBP at 9 months (mmHg) | 131.6 vs 138.2 | -6.1 (95% CI-9.75 to -2.39) * | p = 0.002 |
|  | DBP at 9 months (mmHg) | 76.3 vs 78.0 | -2.9 (95% CI -4.85 to -0.93) * | p = 0.005 |
|  | SBP in minority ethnicity subjects at 9 months (mmHg) | 133.0 vs 140.3 | -6.4 (95% CI -11.16 to -1.68) * | p = 0.009 |
|  | DBP in minority ethnicity subjects at 9 months (mmHg) | 77.9 vs 78.8 | -2.9 (95% CI -5.88 to -0.08) * | p = 0.044 |
| Carter B. (2018), USA (29) | Proportion of patients with uncontrolled hypertension at 12 months (%) | NS | NS |  |
|  | SBP in patients with uncontrolled BP at 12 months (mmHg) | NS | NS |  |
|  | HbA1c in patients with HbA1c ≥ 7.5% at baseline at 12 months (%) | NS | NS |  |
|  | Proportion of patients with uncontrolled diabetes mellitus at 12 months (%) | NS | NS |  |
|  | LDL-C for patients with LDL ≥ 100 mg/dl at baseline at 12 months (mg/dl) | NS | NS |  |
|  | LDL-C for patients with LDL ≥ 130 mg/dl at baseline at 12 months (mg/dl) | NS | NS |  |
|  | Proportion of advising smokers to quit at 12 months (%) | NS | NS |  |
|  | Proportion of patients with diabetic foot examination at 12 months (%) | NS | NS |  |
|  | Proportion of patients with dilated eye examination recommended at 12 months (%) | NS | NS |  |
|  | Proportion of patients with microalbumin order recommended at 12 months (%) | NS | NS |  |
|  | Proportion of patients with Body Mass Index screening and follow-up at 12 months (%) | 68.0 vs 37.4 | **-** | p < 0.001 |
|  | Proportion of patients with alcohol use screening at 12 months (%) | 98.0 vs 88.2 | - | p < 0.001 |
| Chen Z. (2013), USA (30) | **24-hour SBP at 6 months (mmHg)** | **120.4 vs 131.8** | - | p < 0.001 |
|  | Proportion of patients with BP control at 6 months (%) | 75.6 vs 50.0 | - | p < 0.001 |
| Finley P. (2002), USA (41) | **6-months Medication Possession ratio (MPR)** | **0.811 vs 0.659** | **-** | p < 0.005 |
|  | Patient satisfaction survey results at 6 months | Patients in intervention group were more likely to have received an antidepressant previously: 25.0 % vs 19.0 % | - | p = 0.044 |
| Finley P. (2003), USA (42) | **6-months Medication Possession ratio (MPR)** | **NS** | **NS** |  |
|  | Brief Inventory for Depressive Symptoms at 6 months | NS | NS |  |
|  | Work and Social Disability Scale at 6 months | NS | NS |  |
|  | Patient satisfaction survey results at 6 months | Patients in the intervention group expressed greater satisfaction than did control group with the personal nature of care, availability of providers, ability of providers to listen, explanation of why antidepressants were prescribed, explanation of how to take the antidepressants and patient's overall satisfaction with the health maintenance organization | - | p < 0.05 |
| Heisler M. (2012), USA (31) | **SBP at 14 months (mmHg)** | **NS** | **NS** |  |
|  | HbA1c at 12 months (%) | NS | NS |  |
|  | LDL-C at 12 months (mg/dl) | NS | NS |  |
| Hogg W. (2009), Canada (32) | SBP (mmHg) | NS | NS |  |
|  | DBP (mmHg) | NS | NS |  |
|  | HbA1c (%) | NS | NS |  |
|  | Quality of life (Short Form 36 Health Survey) | NS | NS |  |
|  | Proportion of patient with screening for breast cancer (%) | NS | NS |  |
|  | Proportion of patient with screening for cervical cancer (%) | NS | NS |  |
|  | Number of patients with influenza vaccination | - | 0.087 (95% CI 0.012-0.162) ** | p = 0.023 |
|  | Number of patients with screening for colorectal cancer | - | 0.167 (95% CI 0.046-0.288) ** | p = 0.0070 |
|  | Number of patients with hearing examination | - | 0.273 (95% CI 0.106-0.44) ** | p = 0.0016 |
|  | Number of patients with eye examination | - | 0.220 (95% CI 0.076-0.364) ** | p = 0.0029 |
| Jameson J. (2010), USA (33) | **HbA1c at 12 months (%)** | **NS** | **NS** |  |
|  | HbA1c for patients of white race/ethnicity at 12 months (%) | NS | NS |  |
|  | HbA1c for patients of nonwhite race/ethnicity at 12 months (%) | NS | NS |  |
|  | HbA1c for male patients at 12 months (%) | -1.90 vs -0.15 | - | p = 0.03 |
|  | Proportion of patients who achieved at least a 1.0 % decrease in HbA1c at 12 months (%) | 67.3 vs 41.2 | - | p = 0.02 |
|  | Patient who achieved at least a 1.0 % decrease in HbA1c for patients of white race/ethnicity at 12 months (%) | NS | NS |  |
|  | Proportion of patients who achieved at least a 1.0 % decrease in HbA1c for patients of nonwhite race/ethnicity at 12 months (%) | 56.3 vs 22.7 | - | p = 0.03 |
|  | Patient who achieved at least a 1.0 % decrease in HbA1c for female patients at 12 months (%) | NS | NS |  |
|  | Proportion of patients who achieved at least a 1.0 % decrease in HbA1c for male patients at 12 months (%) | 72.0 vs 28.0 | - | p = 0.002 |
| Lenaghan E. (2007), UK (43) | EQ-5d utility score at 6 months | NS | NS |  |
| Omran D. (2015), Canada (34) | **6-months Medication Possession ratio (MPR)** | NS | NS |  |
| Pape G. (2011), USA (35) | Proportion of patients with BP at target goal at 24 months (%) | NS | NS |  |
|  | SBP at 24 months (mmHg) | NS | NS |  |
|  | DBP at 24 months (mmHg) | NS | NS |  |
|  | HbA1c at 24 months (%) | NS | NS |  |
|  | Proportion of patients with HbA1c at target goal at 24 months (%) | NS | NS |  |
|  | **LDL-C at 24 months (mg/dl)** | **83.0 vs 95.0** | **-** | p < 0.001 |
|  | **Proportion of patients with LDL-C at target goal at 24 months (%)** | **78.0 vs 50.0** | **-** | p = 0.003 |
|  | **Proportion of patients with LDL-C at target if they were not at goal baseline at 24 months (%)** | **74.0 vs 48.0** | **-** | p = 0.001 |
|  | Proportion of patients with LDL-C test within the past 12 months at 24 months (%) | 95.0 vs 82.0 | - | p = 0.04 |
|  | Patient satisfaction survey results at 24 months | NS | NS |  |
| Sellors J. (2008), Canada (45) | Quality of life (Short Form 36 Health Survey) at 5 months | NS | NS |  |
| Simpson S. (2011), Canada (36) | **Proportion of patients with diminution of 10.0 % of SBP at 12 months (%)** | **37.0 vs 23.0 (OR 1.91, 95% CI 1.11–3.28)** | **-** | p = 0.02 |
|  | **Proportion of patients with elevated blood pressure at baseline with diminution of 10.0 % of SBP at 12 months (%)** | **50.0 vs 28.0 (OR 2.55, 95% CI 1.30–5.01)** | **-** | p = 0.007 |
|  | **SBP at 12 months (mmHg)** | -7.4 vs -2.5 | **-4.9 (95% CI -8.7 to -1.0) *** | p = 0.002 |
|  | **DBP at 12 months (mmHg)** | -2.3 vs 0.6 | **-2.9 (95% CI -5.6 to -0.2) *** | p < 0.05 |
|  | **SBP for patients with elevated blood pressure at 12 months (mmHg)** | **-13.9 vs -6.7** | - | p = 0.002 |
|  | **HbA1c at 12 months (%)** | **NS** | **-** |  |
|  | **LDL-C at 12 months (mmol/l)** | **NS** | **-** |  |
|  | **HDL-C at 12 months (mmol/l)** | **NS** | **-** |  |
|  | **Total cholesterol at 12 months (mmol/l)** | **NS** | **-** |  |
|  | Predicted 10-year risk of cardiovascular events at 12 months (%) | -2.7 vs -1.2 | -1.5 (95% CI -0.2 to 3.3) * | p = 0.005 |
| Smith S. (2016), USA (37) | Proportion of patients with BP control at 9 months (%) | NS | NS |  |
|  | SBP at 9 months (mmHg) | 132 ± 16 vs 141 ± 20 | -6.62 (95% CI -12.8 to -0.44) * | p = 0.036 |
|  | DBP at 9 months (mmHg) | NS | NS |  |
|  | Proportion of patient reporting high adherence (antihypertensive drugs) at 9 months (%) | NS | NS |  |
|  | Proportion of patient with an improvement from low to high medication adherence (antihypertensive use) at 9 months (%) | NS | NS |  |
|  | Proportion of patients with an improvement from low to high medication adherence minority ethnicity subjects (antihypertensive use) at 9 months (%) | 8.1 vs 0.0 | - | p = 0.016 |
| Tahaineh L. (2011), Jordan (38) | **Proportion of patients reached their LDL-C goal at 6 months (%)** | 94.5 vs 71.2 | - | p < 0.001 |
|  | Proportion of patients reached their total cholesterol goal at 6 months (%) | 87.7 vs 73.1 | - | p = 0.038 |
|  | Proportion of patients reached their HDL-C goal at 6 months (%) | 28.8 vs 50.0 | - | p = 0.016 |
|  | Proportion of patients reached their triglycerides goal of less than 150 mg/dl at 6 months (%) | NS | NS |  |
| Tobari H. (2010), Japan (39) | Proportion of patients with BP control at home at 6 months (%) | NS | NS |  |
|  | SBP at office at 6 months (mmHg) | NS | NS |  |
|  | DBP at office at 6 months (mmHg) | NS | NS |  |
|  | SBP at home at 6 months (mmHg) | NS | NS |  |
|  | DBP at home at 6 months (mmHg) | -3.3 (-4.8 to -1.8) vs -1.4 (-2.9 to 0.1) | -2.8 (95% CI -5.5 to -0.1) * | p = 0.04 |
|  | Proportion of patient with alcohol consumption > 23g/day at 6 months (%) | NS | NS |  |
|  | Proportion of patient with brisk walking > 30 min/day at 6 months (%) | NS | NS |  |
|  | Body Mass Index at 6 months (kg/m²) | -0.4 (-0.7 to -0.2) vs 0.0 (-0.2 to 0.2) | -0.4 (95% CI -0.7 to -0.1) * | p = 0.008 |
|  | Sodium reduction score at 6 months | 1.3 (0.9 to 1.7) vs 0.0 (-0.4 to 0.4) | 1.2 (95% CI 0.5-2.0) ** | p = 0.002 |
|  | Number of smokers at 6 months | 9.0 (14) vs 19.0 (30) | 0.4 (95% CI 0.2-0.9) ** | p = 0.04 |
| Weber C. (2010), USA (40) | **Change in 24-hour SBP at 9 months (mmHg)** | **136.0 to 130.5 vs 135.5 to 121.4** | **-** | p < 0.001 |
|  | **Change in 24-hour DBP at 9 months (mmHg)** | **76.6 to 73.7 vs 76.0 to 69.2** | **-** | p < 0.001 |

Abbreviations: BP, blood pressure ; DBP, diastolic blood pressure ; HbA1c, glycosylated hemoglobin ; HDL-C, high-density lipoprotein cholesterol ; LDL-C, low-density lipoprotein cholesterol ; SBP, systolic blood pressure

Legend: **Bold text: primary outcomes of study ;** * Negative values indicate Intervention Group has larger change ; ** Positive values indicate Intervention Group has larger change
